# Ecological Momentary Assessment of Depression in People With Advanced Dementia: Longitudinal Pilot Study

**DOI:** 10.2196/29021

**Published:** 2021-08-04

**Authors:** Iulia Niculescu, Hannah Quirt, Twinkle Arora, Terry Borsook, Robin Green, Brett Ford, Andrea Iaboni

**Affiliations:** 1 Department of Rehabilitation Sciences University of Toronto Toronto, ON Canada; 2 KITE Toronto Rehabilitation Institute University Health Network Toronto, ON Canada; 3 Department of Psychiatry University of Toronto Toronto, ON Canada; 4 Department of Psychology University of Toronto Toronto, ON Canada

**Keywords:** dementia, depression, ecological momentary assessment, tool performance

## Abstract

**Background:**

Barriers to assessing depression in advanced dementia include the presence of informant and patient recall biases. Ecological momentary assessment provides an improved approach for mood assessment by collecting observations in intervals throughout the day, decreasing recall bias, and increasing ecological validity.

**Objective:**

This study aims to evaluate the feasibility, reliability, and validity of the modified 4-item Cornell Scale for Depression in Dementia for Momentary Assessment (mCSDD4-MA) tool to assess depression in patients with advanced dementia.

**Methods:**

A intensive longitudinal pilot study design was used. A total of 12 participants with advanced dementia were enrolled from an inpatient psychogeriatric unit. Participants were assessed using clinical depression assessments at admission and discharge. Research staff recorded observations four times a day for 6 weeks on phones with access to the mCSDD4-MA tool. Descriptive data related to feasibility were reported (ie, completion rates). Statistical models were used to examine the interrater reliability and construct and predictive validity of the data.

**Results:**

Overall, 1923 observations were completed, representing 55.06% (1923/3496) of all rating opportunities with 2 raters and 66.01% (1923/2913) with at least one rater. Moderate interrater reliability was demonstrated for all items, except for *lack of interest*. Moderate correlations were observed between observers and patient-reported outcomes, where observers reported fewer symptoms relative to participants’ self-reports. Several items were associated with and able to predict depression.

**Conclusions:**

The mCSDD4-MA tool was feasible to use, and most items in the tool showed moderate reliability and validity for assessing depression in dementia. Repeated and real-time depression assessment in advanced dementia holds promise for the identification of clinical depression and depressive symptoms.

## Introduction

### Background

#### Dementia and Depression

Dementia and depression are the most common psychiatric conditions in aging, and there is considerable overlap between them, with the prevalence of depression between 5% and 77% in people with dementia and between 7% and 54% in people at the advanced stage of dementia [1-3]. This wide range demonstrates the challenge in identifying depression in individuals with dementia, including individuals with advanced dementia, a group frequently excluded from studies [3]. The overlap between symptoms of depression and symptoms of dementia (eg, concentration difficulties and apathy) can also confound the diagnosis of depression, making it difficult to assess [4,5]. Many clinical interviews and assessments for depression in dementia include both informant reports and self-reports, and informant reports can be affected by confounding depressive symptoms for symptoms of dementia, mood-congruent biases (eg, related to caregiver burden projected onto the person with dementia), and recall biases [6,7]. Self-reports of people with dementia are limited by memory impairment, poor insight, and language impairment [8-10]. Although validated criteria and tools exist, such as the 19-item Cornell Scale for Depression in Dementia-19 (CSDD-19) [4-11], there is an opportunity to improve the detection and assessment of depression in people with advanced dementia [12,13]. People with dementia and comorbid depression are at risk for negative outcomes, such as hastened cognitive decline and higher rates of morbidities and mortality [14,15]. Detecting depression where it might otherwise be missed provides an opportunity for greatly enhanced patient care in this vulnerable population.

#### Ecological Momentary Assessment

Novel data collection methodologies provide promising opportunities for improving the measurement of depression in people with dementia. Ecological momentary assessment (EMA) encompasses a range of longitudinal data collection methods that capture momentary symptoms repeatedly over time and are typically registered on mobile devices [9]. Real-time and repeated measurements of behaviors and emotions can provide valuable information related to an individual’s dynamic internal state and fluctuations in the expression of symptoms. EMA helps to address various methodological limitations of conventional tools, such as reducing recall bias and enhancing the ecological validity of the data collected [9]. EMA studies in older adults have demonstrated its feasibility, enhanced precision of outcome measurement, and the ability to identify clinically significant depressive symptoms, although most studies exclude people with dementia and are typically self-reported [16-18]. Informant-rated EMA studies are less common than self-reported EMA studies but have been used in the population of people with dementia. For example, daily self-reports of emotional well-being in people with dementia have been compared with informant reports, and internal consistency was found between the two data sources [19]. The use of an observational affect scale was examined in individuals with dementia using EMA. The scale demonstrated excellent reliability among activity therapists as well as family members and nursing assistants and good validity [20]. EMA has thus been used to monitor daily life behaviors and well-being in people with dementia, and these studies have demonstrated the validity of informant ratings and the ability to capture individual differences over time [20-23]. However, no EMA depression screening tools have been developed for people with advanced dementia.

### Objective

This study seeks to address these gaps in a pilot intensive longitudinal EMA study of people with advanced dementia in an inpatient psychogeriatric unit. The aim of this study is to evaluate the psychometric performance of an EMA tool for assessing depression in people with advanced dementia. The first objective is to test the preliminary feasibility outcomes of an observer-rated EMA tool by examining the completion rates and observations of participant acceptability. The second objective is to test the reliability of an observer-rated EMA tool in advanced dementia by examining the reliability of within-person changes and interrater reliability. The third objective is to explore the construct validity and ability of the tool to predict clinical depression and depressive symptoms in patients with advanced dementia. To address these objectives, we conducted a pilot intensive longitudinal study using a modified 4-item Cornell Scale for Depression in Dementia for Momentary Assessment (mCSDD4-MA) tool.

## Methods

### Participants and Sample Size

Participants were patients admitted to the Specialized Dementia Unit at the Toronto Rehabilitation Institute. For study inclusion, participants should be aged ≥65 years and have a diagnosis of moderate-to-severe dementia based on a Mini-Mental State Examination [24] score of <20 [3]. Substitute decision makers provided informed consent, and participants were excluded if they showed signs of dissent to the study procedures, had a previous history of bipolar disorder or schizophrenia, were receiving palliative care, or were unable to understand and speak English (ie, required to self-report).

In keeping with previous pilot EMA studies [16,25,26], the sample comprised 12 participants. Recommendations for determining sample size in intensive longitudinal designs are based on the power of both the within- and between-person sample sizes [27,28]. Despite our smaller between-person sample size (n=12), the within-person sample size (ie, number of repeated observations) is important in detecting the reliability of the random effects and within-person variability and typically requires >50 observations per individual and >1000 observations in total [29-31]. With our study design, we aim to achieve a large number of observations well above this cutoff (ie, eight observations per day for 6 weeks, totaling approximately 336 observations per participant), providing sufficient power for our primary within-person analysis [32]. Our third objective, which involved a between-person analysis, was exploratory in nature and no sample size calculation was completed.

### Design and Setting

We used a pilot observational study design. Observers consisted of 4 trained research staff members. The study was set on the Specialized Dementia Unit at the Toronto Rehabilitation Institute, a psychogeriatric unit caring for people with behavioral and psychological symptoms of dementia. This study was approved by the research ethics board of the University Health Network (Coordinated Approval Process for Clinical Research ID: 19-5132).

### Measures

#### Participant Characterization

At baseline, demographic data collected included sex, age, and dementia diagnosis. The Mini-Mental State Examination was completed by a research assistant to assess cognition [24].

#### Outcome Variables

##### mCSDD4-MA Tool

The mCSDD4-MA tool (Table 1 and Textbox 1) was used as the primary data collection tool. The tool measures depressive symptoms collected by observers, modified for the purposes of this study from the 4-item CSDD (CSDD-4) [13]. Modifications included changing the retrospective language in the CSDD-4 tool to refer to the present, as is necessary for momentary assessments. The final tool consisted of five observational items: *sadness, anxiety, irritability,* and *lack of interest* (ie, from the original tool). *Negativity* was added as it is common in other assessments, including the CSDD-19 tool, and has good specificity in distinguishing between individuals with and without depression in dementia (Table 1) [1,11,33]. In addition to the observational component, a patient-reported component was added, which was unique to the tool (Textbox 1). Patient-reported outcomes included sadness and anxiety as they were central symptoms of depression in older adults [34], were relatively simple concepts to communicate [35], and have shown to be discordant between informants and patients [7].

**Table 1:** Developed observational items in the modified 4-item Cornell Scale for Depression in Dementia for Momentary Assessment tool for people with advanced dementia.

Original CSDD^a^ item	Question	mCSDD4-MA^b^ tool items	Response scale
Introduction	I am going to ask you questions about how your relative has been feeling during the past week.	Looking at the person right now and reflecting on their mood today	N/A^c^
Sadness	Has your relative been feeling down, *sad*, *or blue*^d^ this past week? Has she/he been crying at all? How many days out of the past week has she been feeling like this?	Does the person seem sad or blue?	No sadness Some sadness A lot of sadness Unable to evaluate
Lack of interest	If a pleasant event were to occur today (ie, going out with spouse, friends, or seeing grandchildren), would your relative be able to enjoy it fully, or might his/her mood get in the way of his/her interest in the event or activity? Does your relative’s mood affect any of the following: his/her ability to *enjoy* activities that used to give him/her *pleasure*, his/her surroundings, his/her feelings for family and friends?	Is the person showing enjoyment or pleasure in what is going on around them?	No lack of interest Some lack of interest Lacking a lot of interest Unable to evaluate
Anxiety	Has your relative been feeling *anxious* this past week? Has she/he been *worrying* about things she/he may not ordinarily worry about or ruminating over things that may not be that important? Has your relative had an anxious, tense, distressed, or apprehensive expression?	Does the person seem anxious or worried?	No anxiety Some anxiety A lot of anxiety Unable to evaluate
Irritability	Has your relative felt short-tempered or easily *annoyed* this past week? Has she/he been feeling *irritable*, impatient, or *angry* this week?	Does the person seem irritable, annoyed, or angry?	No irritability Some irritability A lot of irritability Unable to evaluate
Negativity	Has your relative felt *pessimistic or discouraged* about his/her future this past week? Can your relative see his/her situation improving? Can your relative be reassured by others that things will be okay or that his/her situation will improve?	Is the person discouraged or expressing pessimistic or negative thoughts?	No negativity Some negativity A lot of negativity Unable to evaluate

^a^CSDD: Cornell Scale for Depression in Dementia.

^b^mCSDD4-MA: 4-item Cornell Scale for Depression in Dementia for Momentary Assessment.

^c^N/A: not applicable.

^d^Italicization indicates the words that were taken from the original tool and used directly in the 4-item Cornell Scale for Depression in Dementia for Momentary Assessment tool.

**Developed self-reported items in the modified 4-item Cornell Scale for Depression in Dementia for Momentary Assessment tool for people with advanced dementia.**

**4-Item Cornell Scale for Depression in Dementia for Momentary Assessment Tool Patient-Reported Items and Scoring**

- Self-reported sadness
  - Are you feeling sad?
    - Yes
    - No
    - Unable to evaluate
- Self-reported anxiety
  - Are you feeling worried?
    - Yes
    - No
    - Unable to evaluate

Observational items were scored on a 3-point scale where *no*=0, *some*=1, and *a lot*=2. Originally, the CSDD-4 tool included *none*=0, *mild/intermittent*=1, and *extreme*=2 [11,13]. Patient-reported items were scored as yes or no. For the self-report items, raters were encouraged to take time to engage with the participants with the intention of asking these items naturally. Where there would be any inclination toward a yes (ie, including maybe), yes would be chosen, whereas only a clear no was scored as a no in the tool. If participants were asleep or receiving care, raters would select *unable to evaluate* for each item. A total score was generated for items that formed part of the CSDD-4 tool. As the other items were novel in the tool, it was not yet known if these could be included in the total score.

##### Provisional Diagnostic Criteria for Depression of Alzheimer’s Disease

The Provisional Diagnostic Criteria for Depression of Alzheimer’s Disease (PDC-dAD) [4] was used to diagnose clinical depression based on the presence of at least three core symptoms (one of which must be depressed mood or decreased positive affect) within a 2-week period that represented a change from previous functioning. These criteria have been validated in people with dementia. Overall, the findings support the criterion, content, and convergent validity of the PDC-dAD [36]. Specifically, the PDC-dAD has shown greater sensitivity to depression in dementia compared with other common clinical interviews, such as the Diagnostic and Statistical Manual of Mental Disorders [3,4,37]. The PDC-dAD was also able to discriminate group differences on the Hamilton Depression Rating Scale and the Neuropsychiatric Inventory (NPI), highlighting its convergent validity [36].

##### The Improved Clinical Global Impressions Scale

The Improved Clinical Global Impressions (iCGI) scale [38] comprises the 7-item (*normal, not ill at all*=1 to *among the most extremely ill patients*=7) Severity subscale and the 13-item (*ideal improvement*=6 to *maximum deterioration*=−6) Improvement subscale. The iCGI has demonstrated good to excellent interrater reliability (ie, intraclass correlations [ICCs] ranging from 0.62-0.94) and large effect sizes in measuring sensitivity to change (ie, Cohen *d* values of 0.76-1.02) and has been validated in people with depression [38,39].

##### NPI Dysphoria Subscale

The NPI dysphoria item was rated on a 3-item severity scale (*mild*=1, *moderate*=2, and *marked*=3) and a 4-item frequency scale (*occasionally*=1, *often*=2, *frequently*=3, *very frequently*=4). The dysphoria subscale has been shown to correlate significantly with the Hamilton Depression Rating Scale and has shown strength as a stand-alone measure, demonstrating good interrater reliability and strong convergent validity with the CSDD-19 [40]. ICCs by items ranged from 0.54-0.89 [40,41]. The NPI has also been validated in people with dementia and was chosen as it was familiar to clinical staff [42,43].

### Procedures

At baseline and at 6 weeks, diagnostic assessments for depression were completed by a geriatric psychiatrist using the PDC-dAD scale [4], the iCGI scale [38], and the NPI dysphoria subscale [42]. Participants were observed by trained research staff for up to four times a day, 7 days a week, over a 6-week period, and their symptoms were recorded using the mCSDD4-MA tool on a mobile phone.

Before the commencement of data collection, observer training for the research staff was undertaken. This consisted of guidance related to detecting and interpreting depressive symptoms based on affective and behavioral cues and explaining the technical aspects of the mCSDD4-MA tool [20]. Preliminary trial ratings were completed and discussed with raters to ensure that the tool was being used correctly and to improve rater consistency. Four raters recorded depressive symptoms exhibited by participants in pairs on a rotating basis, four times a day (ie, 10-11 AM, 1-2 PM, 4-5 PM, and 7-8 PM) using the tool. The pairs of raters responsible for observing participants on any given day observed all of the enrolled participants within the 1-hour observation period at each timeslot. The raters were blinded to the depression diagnosis for all participants and their co-rater’s depressive symptom ratings.

### Statistical Analyses

A large number of observations (approximately 4 observations × 12 participants × 2 raters × approximately 7 days × approximately 6 weeks) were undertaken. Descriptive analyses were completed for the demographic and EMA data, including feasibility data (ie, completion rates, *unable to evaluate* ratings, and observations of participant acceptability). Completion rates included *unable to evaluate* ratings as completed observations, whereas missing data were defined as the absence of a reported observation during the assigned timeslot. Having reported a participant as unable to be evaluated was thus not classified as a missed observation and instead indicated feasibility data related to observing participants.

Separate cross-classified mixed effects ordinal logistic regression models (ie, cumulative link mixed models) were fit for each item of the mCSDD4-MA tool as the dependent variable, with day and hour variables as fixed effects, participant and observer variables as crossed random effects, and a fixed interaction between day and participant [44]. These models provided estimates of the variances of the random intercepts for participants and observers. The ICC values were generated from these variances [45]. A higher participant ICC would suggest that the variability of the random intercepts was accounted for largely by mood changes in the participants and less because of the sources of error related to the observers [44].

Polychoric correlations (*r*) were generated to examine the interrater reliability between pairs of raters for each item [46]. Krippendorff α values were also generated for each item, given that they evaluate the agreement between multiple raters and multiple time periods and have shown to handle missing data well [47]. Consistent with previous literature, a value of α>.67 is used to denote moderate agreement and α>.80 for excellent agreement [48]. Pairwise polychoric correlations and the level of incongruency between observers and self-reports were generated to examine the relationship between groups of ratings.

To establish construct validity, participants were categorized into clinically depressed and nondepressed groups at baseline, as determined by the PDC-dAD. Total scores for each mCSDD4-MA item and a total score for the baseline week were generated by averaging each participant’s first week data. Wilcoxon rank-sum tests between the 2 groups were run for each item and for the total score, and Cohen *d* effect sizes were generated for each item.

Additional ordinal logistic regression models were fit (ie, cumulative link models) to establish if EMA data could predict clinical depression at the start and end of the study. These models were generated for each item individually, with the mCSDD-4MA symptom ratings and the interaction of the mCSDD-4MA symptom ratings and day inserted as fixed effects. A model was also generated using the total score at each time point and the interaction of the total score and day as fixed effects. The presence of clinical depression on the PDC-dAD admission and discharge assessments was the dependent variable for all models. This process was repeated for the iCGI admission and discharge as dependent variables. All statistical tests were analyzed with *P*>.05.

## Results

### Feasibility and Completion Rates

The demographic characteristics of the participants are presented in Table 2. A total of 1923 observations were completed. This represented a 55.06% (1923/3496) completion rate across the 6-month study, based on 2 raters present at each timeslot, 7 days a week. When excluding weekends and the 7 PM timeslot, the completion rate was 92.01% (1923/2090), with 2 raters present. If at least one rater was present at any point in time, the rate was 66.01% (1923/2913) for 7 days a week. Once weekends and evenings were excluded, the completion rate increased to 98.01% (1923/1962), with at least one rater present. Across the day, 29.02% (558/1923), 31.98% (615/1923), 30.99% (596/1923), and 8.01% (154/1923) of all reported observations occurred at the 10 AM, 1 PM, 4 PM, and 7 PM timeslots, respectively. The majority of the data were skewed toward reporting the absence of symptoms. The most to least frequent items reported were *lack of interest, sadness, anxiety, irritability,* and *negativity* (Multimedia Appendices 1 and 2).

Overall, the rating *unable to evaluate* was selected at 26.99% (519/1923) of the observations, 41.03% (789/1923) of the self-reported sadness, and 43.52% (837/1923) of the self-reported anxiety items. The 7 PM-8 PM timeslot resulted in the greatest inability to evaluate participants where more than half of all observations (83/154, 53.9%) and self-reports during this time were reported as unable to be evaluated, usually because the participants were already asleep. The 10 AM-11 AM timeslot was next, where 32.9% (184/558) of each observational rating could not be evaluated during that time (Multimedia Appendix 3). Overall, participants’ experiences with being assessed were positive, and many expressed appreciations for visits from the observers.

On the basis of the random intercept variances of the participant and the observer, the participant ICCs ranged from 0.13-0.48 for the different symptoms, whereas the observer ICC ranged from 0.00-0.06. Thus, the variability in random intercepts was accounted for primarily by the participants, rather than the rater for most symptoms (Multimedia Appendix 4).

**Table 2:** Demographic characteristics of patient participants (N=12).

Characteristics			Total sample (N=12)		Depressive symptoms (n=4)^a^		No depressive symptoms (n=8)
Age (years), mean (SD)			77.4 (8.2)		81.3 (9.3)		75.5 (6.7)
**Dementia type, n (%)**							
	Alzheimer	9 (75)		3 (75)		6 (75)	
	Vascular	2 (17)		0 (0)		2 (25)	
	Parkinson dementia	1 (8)		1 (25)		0 (0)	
MMSE^b^, median (IQR)			0 (2.5)		0 (4.8)		0 (2.5)
Sex (female), n (%)			5 (42)		3 (75)		2 (25)
Duration in study (days), mean (SD)			38.1 (8.3)		35.5 (11.9)		39.4 (6.4)
**NPI^c^ admission, mean (SD)**			42.3 (22.3)		51.5 (13.6)		37.6 (25.1)
	NPI dysphoria admission	2.83 (4.7)		8.50 (4.1)		0 (0)	
**NPI discharge, mean (SD)**			18.9 (15.3)		24.8 (6.6)		16.0 (18.0)
	NPI dysphoria discharge	2.00 (3.6)		4.00 (4.6)		1.00 (2.8)	
PDC-dAD^d^ depressed admission, n (%)			2 (17)		2 (50)		0 (0)
PDC-dAD depressed discharge, n (%)			1 (8.3)		1 (25)		0 (0)
iCGI^e^ admission, mean (SD)			2.92 (1.4)		4.25 (1.7)		2.25 (0.7)
iCGI discharge, mean (SD)			2.08 (1.2)		2.75 (1.3)		1.75 (1.0)
iCGI improvement score, mean (SD)			1.00 (2.0)		1.50 (3.1)		0.75 (1.4)

^a^Defined by a Neuropsychiatric Inventory cutoff >4.

^b^MMSE: Mini-Mental State Examination.

^c^NPI: Neuropsychiatric Inventory.

^d^PDC-dAD: Provisional Diagnostic Criteria for Depression of Alzheimer’s Disease.

^e^iCGI: Improved Clinical Global Impressions.

### Interrater Reliability

For all pairs of raters, interrater reliability ranged from 0.67-0.92 for *sadness*, 0.57-0.83 for *anxiety*, 0.41-0.90 for *irritability*, −0.07 to 0.82 for *negativity*, and 0.24-0.79 for *lack of interest* (Table 3). These analyses identified that the fourth rater was consistently less reliable, given the differences in their scores. Thus, separate reliability analyses were conducted using all raters and only raters 1-3.

Krippendorff α values across all raters were generated and showed moderate reliability for *sadness* (α=.74) and *irritability* (α=.67) but lower reliability for *negativity* (α=.62), *anxiety* (α=.61), and *lack of interest* (α=.45). Once the fourth rater was excluded, the α values increased, but the trends remained the same (Table 4).

**Table 3:** Polychoric correlations (*r*) of the observational data comparing pairs of the 4 researchers for each of the items.

Raters		1	2	3
**Sadness**				
	2	0.91	—^a^	—
	3	0.86	0.67	—
	4	0.75	0.59	0.57
**Irritability**				
	2	0.87	—	—
	3	0.90	0.72	—
	4	0.66	0.50	0.41
**Negativity**				
	2	0.75	—	—
	3	0.82	0.62	—
	4	0.28	−0.07	0.71
**Anxiety**				
	2	0.82	—	—
	3	0.83	0.71	—
	4	0.75	0.59	0.57
**Lack of interest**				
	2	0.69	—	—
	3	0.79	0.50	—
	4	0.34	0.27	0.24

^a^N/A: not applicable.

**Table 4:** Krippendorff α values for ecological momentary assessment item data by research staff.

Item		Krippendorff α
**Raters 1-4**		
	Sadness	.74
	Anxiety	.61
	Irritability	.67
	Lack of interest	.45
	Negativity	.62
**Raters 1-3**		
	Sadness	.78
	Anxiety	.65
	Irritability	.77
	Lack of interest	.54
	Negativity	.62

### Concordance Between Observational and Self-reported Items

Patient–self-reported symptoms were moderately correlated with observer-rated sadness (*r*=0.68) and anxiety (*r*=0.57). When participants reported feeling sad or anxious, raters would observe sadness 88.1% (730/829) of the time and would observe anxiety 78.9 % (601/761) of the time. When raters reported observed depressive symptoms, participants would confirm feeling sad in 90.97% (968/1064) of the cases and would confirm feeling worried in 93.83% (1081/1152) of the cases. Overall, 72.95% (1403/1923) of the ratings showed agreement between observers and self-reports of sadness and anxiety (Multimedia Appendix 5).

### Construct Validity

Observer-rated *sadness*, *anxiety*, and total symptom score in the first week of assessment were significantly associated with the presence of clinical depression at baseline, as determined by the PDC-dAD (Wilcoxon-rank sum, W=20, *P*=.04, Cohen *d*=1.00 for *sadness*; W=20, *P*=.04, Cohen *d*=0.49 for *anxiety*; and W=20, *P*=.03, Cohen *d*=1.00 for the total score).

Observational and self-reported measures of *sadness* and *anxiety* over the course of the study were associated with clinical depression diagnosis over time, as determined by the PDC-dAD at baseline and at 6 weeks. Scoring at least *some* (vs no) observational *sadness* and *anxiety* increased the log odds of clinical depression diagnosis by 2.74 and 1.51, respectively. Likewise, scoring *a lot* (vs *no*) of observational *sadness* and *anxiety* increased the log odds of clinical depression diagnosis by 5.37 and 3.13, respectively. Finally, answering *yes* (vs *no*) on the sadness and anxiety self-reports increased the log odds of clinical depression diagnosis by 2.20 and 2.58, respectively (Table 5).

**Table 5:** Association between items in the modified 4-item Cornell Scale for Depression in Dementia for Momentary Assessment tool and clinical depression, as determined by the Provisional Diagnostic Criteria for Depression and Dementia, over the course of the study.

Items and item score			Estimate (SE)		*P* value		95% CI
**Sadness**							
	2	2.74 (0.62)		<.001^a^		1.52 to 3.95	
	3	5.37 (0.73)		<.001^a^		3.93 to 6.80	
**Anxiety**							
	2	1.51 (0.32)		<.001^a^		0.87 to 2.15	
	3	3.13 (0.58)		<.001^a^		2.00 to 4.26	
**Irritability**							
	2	0.44 (0.62)		.47		−0.77 to 1.67	
	3	0.61 (0.79)		.44		−0.95 to 2.17	
**Lack of interest**							
	2	−0.46 (0.54)		.40		−1.52 to 0.60	
	3	0.74 (0.85)		.86		−0.94 to 2.42	
**Negativity**							
	2	0.74 (0.58)		.20		−0.39 to 1.88	
	3	1.61 (1.93)		.40		−2.16 to 5.40	
**Self-reported sadness**							
	2	2.20 (0.47)		<.001^a^		1.07 to 2.94	
**Self-reported anxiety**							
	2	2.58 (0.51)		<.001^a^		1.59 to 3.58	

^a^*P*=.04.

In addition to *sadness*, *anxiety*, and self-reported anxiety, *negativity* over the course of the study also predicted depressive symptom severity, as measured by the iCGI Severity scale. Scoring *a lot* of *sadness* and *anxiety* relative to *no* increased the log odds of severe depressive symptoms by 4.49 and 4.81, respectively. Scoring *some anxiety* and *negativity* compared with *no* increased the log odds of severe depressive symptoms by 1.93 and 1.13, respectively. Finally, answering *yes* compared with *no* for the anxiety self-report decreased the log odds of severe depressive symptoms by 0.63 (Multimedia Appendix 6).

The total CSDD-4 score generated at each observation point did not predict clinical depression diagnosis or depressive symptoms as determined by the PDC-dAD or iCGI over the course of the study.

## Discussion

### Principal Findings

Our study evaluated the performance of the mCSDD4-MA tool for assessing depression in people with advanced dementia. EMA ratings of depressive symptoms show potential for identifying clinical depression and can contribute to a wider understanding of depression assessment in this population. EMA approach showed preliminary feasibility, and the items demonstrated moderate reliability, with the exception of *lack of interest*. Moderate correlations were observed between the observational and patient-reported items. In addition, the tool showed construct validity across several items and for the total score and promising predictive validity for several items.

The mCSDD4-MA tool was feasible and acceptable to the participants, with the participants enjoying engagement by the research staff. Overall, 7 PM-8 PM and 10 AM-11 AM timeslots accounted for the lowest proportion of observations based on both observer completion rates and their ability to observe participants. In terms of observing participants, these times may occur when participants are sleeping or receiving personal care. From a feasibility perspective, it may be appropriate to cut down to 2 observations per day in the afternoon. However, the next steps require comparing the sensitivity of the tool when observing participants two times versus four times a day to conclude if two observations are sufficient.

Capturing observational ratings of depressive symptoms repeatedly in real time was found to be a reliable method for assessment. Item-level analyses demonstrated that *sadness* and *irritability* were the most reliable and that *anxiety* and *negativity* were less reliable. This is consistent with previous research in which observers who repeatedly rated effect in people with dementia in real time found high interrater reliabilities for sadness and irritability [20]. Sadness and irritability may be easily recognizable because of their intensity and are thought to be biologically hard-wired emotions [20,49]. Ratings of *anxiety* were less reliable between raters, which may be related to their high heterogeneity in the presence of emotional disorders [20].

Although four out of five items demonstrated good psychometric properties, *lack of interest* displayed clear psychometric problems for which there are several possible explanations. These relate to the time taken to assess the item, the definition of the item, and the overlap of *lack of interest* with apathy. First, it is possible that insufficient time was spent observing participants to properly assess their degree of interest. The evaluation of interest requires both the presence of engaging activities to stimulate interest as well as the time to observe whether an individual is deriving any enjoyment from the activity [20]. Even in a well-resourced inpatient unit, there may still be moments throughout the day of low activity or understimulation for participants. Second, the adaptation of the *lack of interest* item for real-time assessment was: “Is the person showing *enjoyment* or *pleasure* in what is going on around them?” with options, “No lack of interest,” “Some lack of *interest*,” and “Lacking a lot of *interest*.” Studies have shown that although pleasure and interest are highly correlated, there is heterogeneity in the definition of anhedonia [50]. As pleasure and enjoyment were included in the question, and interest was used in the response, this may have affected the understanding of the item. Finally, symptom overlap with apathy (ie, loss of interest and motivation, fatigue, and low social engagement) may have confounded the item [51]. Overall, there is a need to develop a more reliable *lack of interest* item for real-time assessment. This would require modifications such as wording the item to be more closely related to the concept of anhedonia and more distinct from apathy, recommending longer observation periods for evaluating the presence of symptoms, and improving rater training [20,52].

Using EMA to measure depressive symptoms in advanced dementia also shows construct and predictive validity, as demonstrated by its association with depression at baseline and over time. Our analyses confirmed the validity of several items, including observed sad and anxious affect, which have been previously reported to predict and correlate with depression and depressive symptoms in people with dementia [19,20]. In this study, we were also able to demonstrate a relationship between patient-reported symptoms in a population with advanced dementia and clinical depression and symptoms. This is a unique finding, as self-reporting is not typically included in observer-rated depression assessments. This lends some support to the inclusion of patient self-reports, in keeping with patient-centered care approaches. *Negativity* was also shown to be associated with depressive symptoms; however, the rating of *negativity* was contingent on the participants’ ability to communicate negative cognitions. Although *negativity* is a highly specific depressive symptom in advanced dementia, it has poor sensitivity given its low frequency. Overall, several items in the mCSDD4-MA tool demonstrated a promising ability to detect clinically significant depression and depressive symptoms.

Discrepancies between informant and patient-reported symptoms are well documented in the literature and were found in this study, illustrating the importance of collecting both types of reports. Low patient-proxy agreement in mood can be attributed to subjectivity in observing these items and raters attributing depressive symptoms to dementia or vice versa [10,53,54]. In this study, the majority of ratings (1403/1923, 72.95%) completed by participants and observers were concordant. In 57% (12/21) and 78% (21/27) of the discordant ratings, the participants self-reported the presence of sad and anxious mood, respectively, whereas observers rated the symptoms as absent. This differs from the literature in which people with dementia have reported *fewer* symptoms than their informants, although some studies have shown similar results [7,8]. Again, this underscores the importance of including patient-reported ratings, although it is important to ensure the reliability of these self-reports. In this study, the severity of cognitive impairment may have affected the reliability of patient-reported outcomes. Some participants agreed to feeling sad or anxious, despite not showing any outward sign of negative affect, leading the observers to doubt whether the participants had understood the question. Thus, there is a need to improve the reliability of self-reports, which could be done by combining some neutral and positively worded questions, in addition to the questions about symptoms to ascertain the consistency of the responses [35].

This study had several limitations. As this was a pilot study, the between-person sample size affected the power and generalizability of the results to a larger population of people with advanced dementia. However, we aimed to compensate for this by achieving a large within-person sample size. In addition, although intensive longitudinal designs are limited in their generalizability to other individuals, they are strengthened by their ability to generalize across situations within individuals [32]. Although certain patient-related (ie, cognitive impairment and level of awareness) and observer-related (ie, quality of training and internal mood states) factors can have an impact on the interpretation of mood, our study did not specifically examine these effects on depression ratings. Future studies can address the psychometric issues with the assessment of interest in people with dementia in real time and develop EMA protocols to improve the overall psychometric properties of the tool. Given the previous findings on caregiver biases, it is important to note that research staff ratings may differ from caregiver ratings, which may limit the generalizability of these findings [6,7]. Therefore, future studies should also examine the performance across different categories of observers.

### Conclusions

A modified CSDD4-MA tool for momentary assessment of depression in people with advanced dementia is feasible and has moderate reliability and validity. Repeated and real-time assessment of mood in these individuals holds promise to monitor depressive symptoms and clinical depression.

## Appendix

Multimedia Appendix 1 Frequency of research staff observations for the observational 4-item Cornell Scale for Depression in Dementia for Momentary Assessment items.

Multimedia Appendix 2 Frequency of the self-reported 4-item Cornell Scale for Depression in Dementia for Momentary Assessment items.

Multimedia Appendix 3 Percentage of data (%) that was rated as unable to be evaluated at each observation period.

Multimedia Appendix 4 Ratios of variance components of the participant and observer variables in the 4-item Cornell Scale for Depression in Dementia for Momentary Assessment items.

Multimedia Appendix 5 The level of congruence between observational sadness and anxiety and self-reported sadness and anxiety.

Multimedia Appendix 6 Association between items in the 4-item Cornell Scale for Depression in Dementia for Momentary Assessment tool and clinical depressive symptoms, as measured by the Improved Clinical Global Impressions scale over the course of the study.

## Abbreviations

CSDD: Cornell Scale for Depression in Dementia
CSDD-4: 4-item Cornell Scale for Depression in Dementia
EMA: ecological momentary assessment
ICC: intraclass correlation
iCGI: Improved Clinical Global Impressions
mCSDD4-MA: 4-item Cornell Scale for Depression in Dementia for Momentary Assessment
NPI: Neuropsychiatric Inventory
PDC-dAD: Provisional Diagnostic Criteria for Depression of Alzheimer’s Disease
