# Supplementary figures and images for: Ecological Momentary Assessment of Depression in People With Advanced Dementia: Longitudinal Pilot Study
Source: JMIR Aging. 2021 Aug 4;4(3):e29021. doi: 10.2196/29021 (PMC8374663; doi:10.2196/29021)

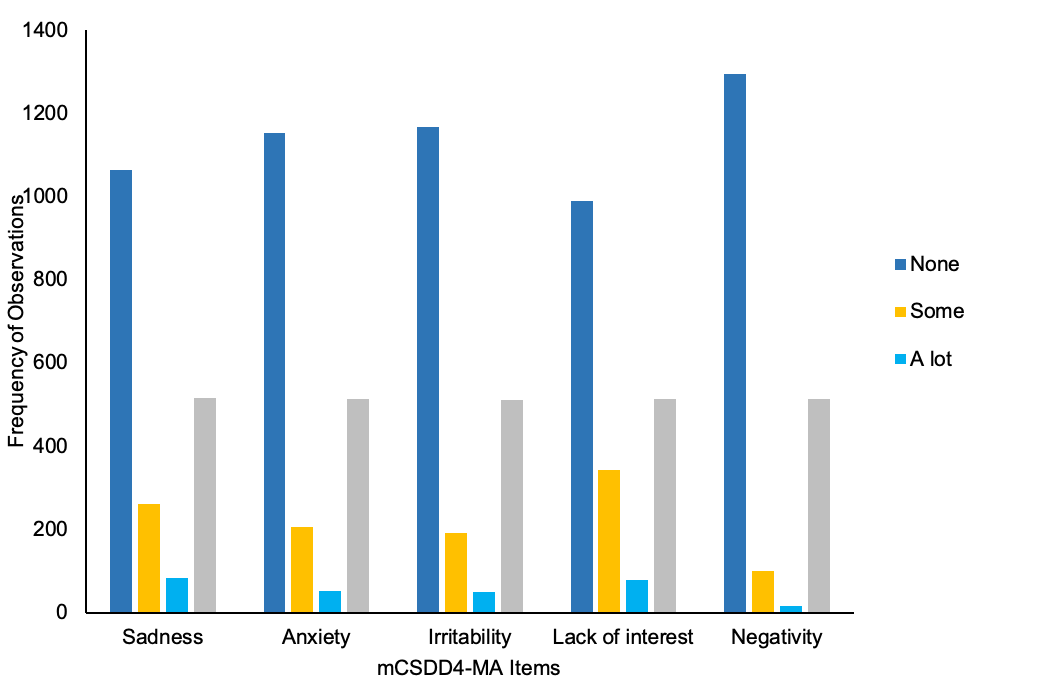

Supplement: Multimedia Appendix 1 [file aging_v4i3e29021_app1.png]

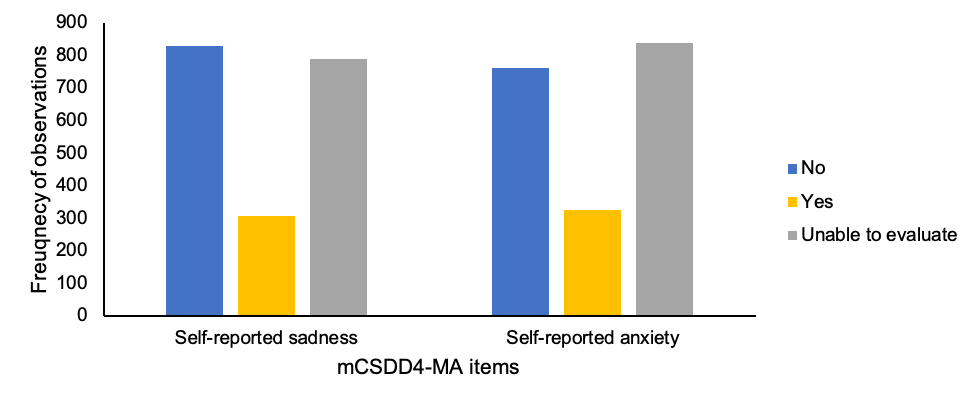

Supplement: Multimedia Appendix 2 [file aging_v4i3e29021_app2.png]

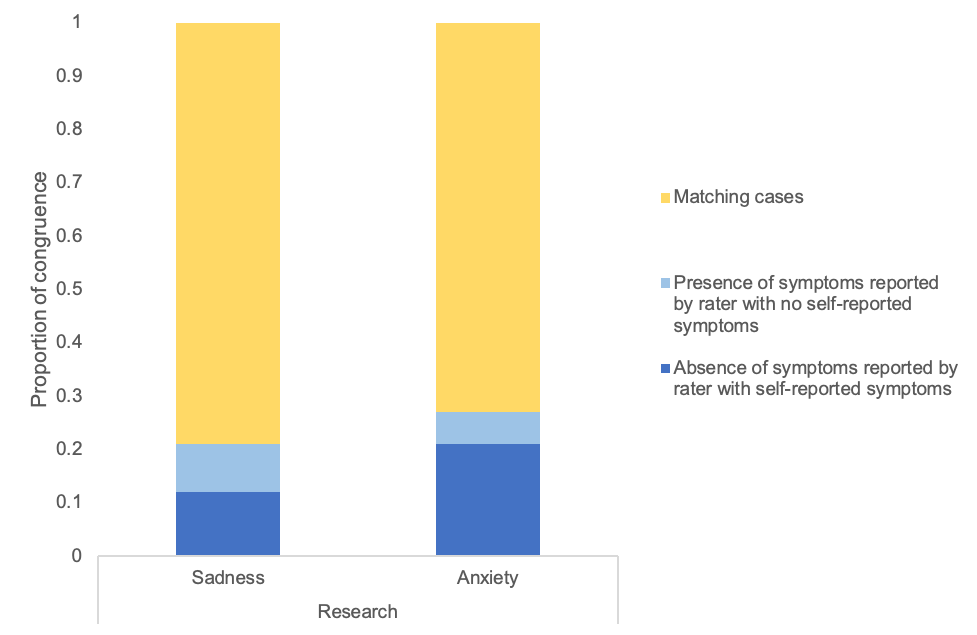

Supplement: Multimedia Appendix 5 [file aging_v4i3e29021_app5.png]
