# Supplementary material for: Ecological Momentary Assessment of Depression in People With Advanced Dementia: Longitudinal Pilot Study
Source: JMIR Aging. 2021 Aug 4;4(3):e29021. doi: 10.2196/29021 (PMC8374663; doi:10.2196/29021)
Supplement: Multimedia Appendix 3 [file aging_v4i3e29021_app3.docx]

Multimedia Appendix 3

**Table S3**. Percent of data (%) that was rated as “unable to be evaluated” at each observation period.

|  | Observational items | Self-reported sadness | Self-reported anxiety |
| --- | --- | --- | --- |
| 10-11am | 33 | 42 | 45 |
| 1-2pm | 28 | 43 | 43 |
| 4-5pm | 13 | 33 | 36 |
| 7-8pm | 54 | 64 | 68 |
