# Supplementary material for: Ecological Momentary Assessment of Depression in People With Advanced Dementia: Longitudinal Pilot Study
Source: JMIR Aging. 2021 Aug 4;4(3):e29021. doi: 10.2196/29021 (PMC8374663; doi:10.2196/29021)
Supplement: Multimedia Appendix 4 [file aging_v4i3e29021_app4.docx]

Multimedia Appendix 4

**Table S4.** Ratios of variance components of participant and observer variables in the mCSDD4-MA items.

| Items | Estimated Level-2 Random Intercept Variance of Participant | Estimated Level-2 Random Intercept Variance of Observer | ICC Participants* | ICC Observers** |
| --- | --- | --- | --- | --- |
| Sadness | 3.08 | 0.12 | 0.48 | 0.04 |
| Anxiety | 2.98 | 0.15 | 0.48 | 0.04 |
| Lack of interest | 0.51 | 0.20 | 0.37 | 0.00 |
| Irritability | 1.90 | 0.01 | 0.35 | 0.02 |
| Negativity | 1.76 | 0.06 | 0.13 | 0.06 |
| Self-reported sadness | 1.50 | 0.01 | 0.31 | 0.00 |
| Self-reported anxiety | 1.46 | 0.02 | 0.31 | 0.01 |

*ICC: Intra-class correlations referring to the Level-2 variance of random-intercepts of participants relative to sum of Level-1 variance of the underlying latent response tendency and the Level-2 variance of random effect of participants

**ICC: Intra-class correlations referring to the Level-2 variance of random-intercepts of observers relative to sum of Level-1 variance of the underlying latent response tendency and the Level-2 variance of random effect of observers
