# Supplementary material for: Ecological Momentary Assessment of Depression in People With Advanced Dementia: Longitudinal Pilot Study
Source: JMIR Aging. 2021 Aug 4;4(3):e29021. doi: 10.2196/29021 (PMC8374663; doi:10.2196/29021)
Supplement: Multimedia Appendix 6 [file aging_v4i3e29021_app6.docx]

Multimedia Appendix 6

**Table S6.** Association between Items in the mCSDD4-MA tool and clinical depressive symptoms as measured by the iCGI over the course of the study

| Items | Item  Score | Estimate | SE | *p* | 95% CI | |
| --- | --- | --- | --- | --- | --- | --- |
|  |  |  |  |  | Lower | Upper |
| Sadness | 2 | 0.65 | 0.35 | 0.06 | -0.03 | 1.34 |
|  | 3 | 4.49 | 0.56 | 0.00* | 3.39 | 5.58 |
| Anxiety | 2 | 1.93 | 0.34 | 0.00* | 1.26 | 2.60 |
|  | 3 | 4.81 | 0.86 | 0.00* | 3.14 | 6.49 |
| Irritability | 2 | 0.36 | 0.30 | 0.24 | -0.24 | 0.97 |
|  | 3 | 0.77 | 0.60 | 0.20 | -0.41 | 1.95 |
| Lack of interest | 2 | 0.03 | 0.27 | 0.90 | -0.49 | 0.55 |
|  | 3 | 0.16 | 0.50 | 0.75 | -0.82 | 1.13 |
| Negativity | 2 | 1.13 | 0.44 | 0.01* | 0.26 | 2.00 |
|  | 3 | 3.39 | 1.78 | 0.06 | -0.01 | 0.00 |
| Self-reported sadness | 2 | -0.03 | 0.30 | 0.91 | -0.63 | 0.56 |
| Self-reported anxiety | 2 | 0.63 | 0.31 | 0.04* | 0.03 | 1.24 |

* denotes *p* < 0.05. SE: Standard error, CI: Confidence interval.
